# Predicting haplotype carriers from SNP genotypes in *Bos taurus* through linear discriminant analysis

**DOI:** 10.1186/s12711-015-0094-8

**Published:** 2015-02-05

**Authors:** Stefano Biffani, Corrado Dimauro, Nicolò Macciotta, Attilio Rossoni, Alessandra Stella, Filippo Biscarini

**Affiliations:** IBBA-CNR, Via Einstein, Loc. Cascina Codazza, Lodi, 26900 Italy; Department of Bioinformatics, PTP, Via Einstein - Loc. Cascina Codazza, Lodi, 26900 Italy; Dipartimento di Agraria, Università di Sassari, Via De Nicola, 9, Sassari, 07100 Italy; ANARB, Italian, Loc. Ferlina, 204, Bussolengo (VR), 37012 Italy

## Abstract

**Background:**

SNP (single nucleotide polymorphisms) genotype data are increasingly available in cattle populations and, among other things, can be used to predict carriers of specific haplotypes. It is therefore convenient to have a practical statistical method for the accurate classification of individuals into carriers and non-carriers. In this paper, we present a procedure combining variable selection (i.e. the selection of predictive SNPs) and linear discriminant analysis for the identification of carriers of a haplotype on BTA19 (*Bos taurus* autosome 19) known to be associated with reduced cow fertility. A population of 3645 Brown Swiss cows and bulls genotyped with the 54K SNP-chip was available for the analysis.

**Results:**

The overall error rate for the prediction of haplotype carriers was on average very low (∼≤1*%*). The error rate was found to depend on the number of SNPs in the model and their density around the region of the haplotype on BTA19. The minimum set of SNPs to still achieve accurate predictions was 5, with a total test error rate of 1.59.

**Conclusions:**

The paper describes a procedure to accurately identify haplotype carriers from SNP genotypes in cattle populations. Very few misclassifications were observed, which indicates that this is a very reliable approach for potential applications in cattle breeding.

## Background

Nanotechnology applications such as next-generation sequencing [1], microarray genotyping [2] and genotyping-by-sequencing [3] generate increasing amounts of single nucleotide polymorphisms (SNP) data. This has a profound impact on modern agriculture, specifically in animal genetics and breeding [4]. Several SNP panels have been designed for the major livestock species [5] and are available at increasingly lower prices. For *Bos taurus* (cattle) only, there are as many as six commercial SNP chips [6]. This wealth of genomic information (“big data”) has prompted the use of advanced bioinformatics and statistical techniques for data storage, manipulation and analysis in animal genetics [7]. The combination of large amounts of SNP data and sophisticated analytical and computation methods has given rise to a range of applications in animal genetics such as, for instance, genome-wide association studies (GWAS) for monogenic and complex traits [8], genome-based prediction of breeding values (“genomic selection”) [9], or the investigation of relationships between populations and their evolutionary history [10].

One application is the prediction of haplotypes or gene alleles from marker genotypes. In humans, for instance, some alleles of the HLA (Human Leukocyte Antigen) complex [11] play an important role in the evaluation of organ transplantation compatibility. Haplotype or gene allele prediction is relevant also in livestock animal species. In dairy cattle, alleles at the *K-casein* locus determine the casein isoforms that will be synthesized, which are associated with cheese yield and quality [12]. Again in cattle, specific haplotypes are known to be associated with embryo losses or perinatal mortality [13]. Thus, it is valuable for cattle selection programmes to identify accurately which haplotypes or alleles the animals carry (and can transmit to the offspring). Traditionally, specific laboratory assays are used to detect gene alleles (e.g. either microsatellite markers [14] or, in cows, directly the casein isoforms in milk samples [15], for the casein example mentioned above). Haplotypes can be reconstructed *in silico* from marker genotypes using family and/or population information [16,17] from which identical-by-descent (IBD) probabilities can be computed to track co-inherited adjacent marker alleles. These approaches usually require extended pedigree information, which is not always available. Also, IBD calculations can become impractical -or even hardly feasible- with high-density SNP-chip data. Especially for less related individuals, a proportion of genotypes may be left unphased thus reducing the accuracy of haplotype reconstruction. More sophisticated techniques do exist that increase the reliability of reconstructed haplotypes and computation efficiency [18], but may not be implementated straightforwardly. Therefore, since allele detection and haplotype reconstruction can be expensive, time-consuming and computationally demanding, it would be convenient to use SNP data—which are often already available for other purposes, e.g. genomic selection—to predict directly haplotypes or gene alleles of interest.

Previous studies on the prediction of haplotypes or gene alleles using SNP data in cattle have been reported: for instance, Pirola et al. [19] used SNP genotypes together with pedigree records to predict *K-casein* alleles in a robust combinatorial formulation of the problem.

In this paper, we describe the use of linear discriminant analysis (LDA) for the prediction of specific haplotype carriers from SNP data. Linear discriminant analysis is a powerful classification technique that is suited when classes are well-separated and predictors have a common covariance structure [20]. LDA has been successfully applied to bovine breed assignment for traceability purposes [21]. Here we present a classification procedure that combines variable selection (i.e. the selection of which predictive SNP loci to include) and linear discriminant analysis for the accurate identification of haplotype carriers. A haplotype known to be linked to reduced cow fertility in a population of Brown Swiss cattle was used to test the methodology.

## Methods

### Experimental data

SNP genotypes and haplotype information were available for a dairy cattle population of 3645 Italian Brown Swiss cows (1476) and bulls (2169; see Table 1).

**Table 1:** **Description of the experimental Brown Swiss cattle population and SNP marker genotypes**

*Number of individuals*		3645
	Haplotype carriers	513
	Non-carriers	3132
	Bulls (carriers)	2169 (323)
	Cows (carriers)	1476 (190)
*Number of SNPs*	54K SNP-chip v2	54609
	7K SNP-chip (extracted)	6858
*Number of SNPs on BTA 19*		
	54K SNP-chip	1512
	7K SNP-chip	211

All animals were genotyped with the BovineSNP50 v2 (54K) Illumina BeadChip. From this chip, the 6858 SNPs corresponding to the Illumina BovineLD BeadChip (7K) were extracted. In this way, all animals had data for both the 54K and 7K (extracted) SNP-chips. Only SNPs on BTA19 (*Bos taurus* autosome 19) were used for the analysis. A total of 1512 and 211 SNPs were located on BTA19 with the 54K and 7K SNP chips, respectively. No individual animal had a call-rate lower than 95*%*. SNPs with a call-rate lower than 95*%* were removed from the analysis. The remaining missing SNP genotypes were imputed based on linkage disequilibrium [22]. Editing for allele frequency was performed within the variable selection procedure (see next section).

The chosen haplotype was BH2, that is ∼909 kb long and is located at the beginning of BTA19 (10.140 - 11.049 Mb). BH2 was previously reported to be associated with stillbirth and calf survival rate [23]. Animals were identified as carriers (coded as 1) or not (coded as 0) of the haplotype. Since the causal mutation contained in the BH2 haplotype is still not known, the status of the animals was based on the haplotype and can be prone to errors with respect to carrying or not the underlying mutation. There were 513 carriers (14.1*%*) and 3132 non-carriers (85.9*%*). Haplotype carriers were evenly distributed between bulls (323, 14.9*%*) and cows (190, 12.9*%*). Haplotypes were inferred from previously phased SNP genotypes based on allele frequency and linkage disequilibrium [22,24]. Phased SNP genotypes on BTA19 that matched the haplotype described by Schwarzenbacher et al. [23] were used to identify carriers.

Data for the present study were provided by the Italian Brown Swiss Breeders Association (ANARB, www.anarb.it).

### Identification of haplotype carriers

Two complementary multivariate statistical tecniques were used for the identification of haplotype carriers: Backward Stepwise Selection (BSS) to select the SNPs that best fit the model, and Linear Discriminant Analysis (LDA) to classify observations, based on the selected SNPs, into carriers and non-carriers.

In order to explore the minimum-sized set of SNPs that still correctly identifies haplotype carriers, different proportions of SNP markers were tested: 2.5,10,15,30,50 and 100*%* of the total number of SNPs for both the 7K and 54K chips. For each proportion of SNPs, BSS and LDA were applied, and the classification error rate was estimated in a 10-fold cross-validation scheme. This process was repeated 100 times -for each proportion of SNPs- each time sampling a different subset of markers (e.g. at each of the 100 repetitions with say 10*%* of the markers, a different 10*%* of the SNPs were randomly selected: this random SNP sample was used to run BSS and LDA in the 10-fold cross-validation routine).

#### Variable selection

Backward stepwise selection (BSS) is a method especially designed for coping with variable selection. It is applied to define the subset of variables that best fit the model. In the present work, BSS was used to select a subset of SNPs that best discriminate among groups. In particular, the minimum number of SNPs that still efficiently discriminate between haplotype carriers and non-carriers and correctly assign new observations was determined.

The BSS model was built step-by-step. In the first step, all *m* SNPs were used and the *m*−1 SNPs that contributed most to correct group assignment were selected. In the following steps, the least relevant SNP was iteratively removed from the model, one at a time, until the SNP subset that best fitted the data was identified. Best fit was defined as the model with the lowest residual sum of squares in a linear regression model. Details of the algorithm can be found in James et al. [25].

The BSS algorithm described above was applied to each tested proportion of SNPs (see Table 2).

**Table 2:** **Procedure** ***HaplotypeCarrierIdentification(P,C)***

**Step**	**Action**
1	**foreach** proportion *P* and SNP-chip *C* (7*K* and 54*K*) **do**
2	**for** *n*=1⋯100**do**
3	randomly split the data into 10 subsets of roughly equal size (*S*={1:10})
	[*10-fold cross-validation*]
4	**for** *k*=1⋯10**do**
5	use *s*:*s*≠*k* subsets from *S* to train the model and subset *k* for validation;
6	in the training set:
7	- delete monomorphic and collinear SNPs;
7	- select the best combinations of SNPs using BSS until *p*⊂*P* SNPs are left;
7	- use BSS-selected SNPs to classify haplotype carriers with LDA;
7	- save SNP discriminant coefficients;
7	- compute the average training error rate;
8	in the validation set:
9	- use BSS-selected SNPs and their discriminant coefficients to classify haplotype carriers;
9	- compute the average validation error rate;

#### Classification

Linear discriminant analysis (LDA) is a multivariate statistical technique whose main objective is to assess how good any specific classification in *k* distinct groups is, given a specific set *P* of *m* predictors. LDA is a supervised statistical learning technique which –unlike unsupervised methods such as cluster analysis– makes use of *a priori* knowledge on which observation belongs to which group. LDA tests the hypothesis of whether groups can be reconstructed based on the available set *P* of predictors. In the present study, the predictors were SNP genotypes and the two groups in which to classify observations were carriers or non-carriers of the BH2 haplotype on BTA19 associated with reduced cow fertility. LDA involves the determination of a linear equation –the discriminant function (DF)– used to predict which group an observation belongs to. The general form of the discriminant function is a linear combination of the *P* predictors:

(1) $$ DF=f(P)=d_{1}P_{1}+d_{2}P_{2}+\dotsb+d_{m}P_{m}   $$

where for *i* in {1:*m*} the *d*_*i*_ are the discriminant coefficients of *DF* and *P*_*i*_ are the values of the *m* predictors involved. Group centroids are then obtained by averaging *f(P)* within groups. The larger the difference between group centroids, relative to the grand centroid (the average of *f(P)* over all groups), the greater the discriminant power. The discriminant coefficients *d*_*m*_ are thus chosen to minimize the within-group variation (around the group centroids) and, at the same time, maximize the between-group variation (around the grand centroid). This is achieved by maximizing -through matrix differentiation- the ratio $\lambda = \frac {\mathbf {d^{\prime }Bd}}{\mathbf {d^{\prime }Wd}}$, where **B** is the between-group (co)variance matrix and **W** is the within-group (co)variance matrix. Given *k* groups, *k*−1 discriminant functions are defined. In the present study, *k*=2 (carriers/non-carriers) and only one discriminant function is defined. The criterion to classify observations into one of the two groups is obtained by applying Equation 1 to each individual observation which results in a value called the discriminant score. An observation is assigned to a specific group if its discriminant score is lower than the cutoff value obtained by calculating the weighted mean distance among group centroids [26].

### Prediction accuracy

In order to estimate the discriminant power of LDA and identify the minimum number of SNPs required to correctly identify haplotype carriers, a 10-fold cross-validation procedure was adopted. Data were split in 10 subsets of approximately equal size. The first subset was treated as validation set, while the model was fit on the remaining nine subsets (the training set). Prior to fitting the model, monomorphic and collinear (correlation > 0.99) SNPs were edited out of the training set, to remove non-informative and redundant predictors and avoid problems due to linear dependencies. This procedure was repeated 10 times, using a different subset each time as validation set. For each fold, the error rate (*ER*) was estimated both in the training and validation set.

The overall 10-fold cross-validation (CV) error in both the training and validation sets was then estimated by averaging the individual-fold error rates:

(2) $$  \overline{CV}=\frac{1}{10} \sum\limits_{i=1}^{10} {ER}_{i}  $$

For each SNP panel (7K and 54K), six proportions of SNPs were tested to estimate the classification error rate on both the training and validation sets. Each 10-fold CV was repeated 100 times per proportion, eventually generating 6000 replicates per SNP-chip (10-fold × 100 repetitions × 6 proportions).

To recap, the whole-procedure is summarised in Table 2.

Three different error rates were measured: (1) the Total Error Rate (TER) defined as the total number of misclassifications over the total sample size; (2) the False Positive Rate (FPR) defined as the number of non-carriers misclassified as carriers over the total number of non carriers; and (3) the False Negative Rate (FNR) defined as the number of carriers misclassified as non-carriers over the total number of carriers.

### Comparison with another classification method

The linear discriminant classifier in Equation 1 was compared to a logistic regression model for binary outcomes. The probability of carrying the haplotype given the SNP genotypes (*P*(*Y*=1|*X*)=*p*(*x*)) was modeled as a linear combination of the predictors (SNPs) through a *logit* link-function in a generalised linear model:

(3) $$  logit(p(\mathbf{x}_{i})) = log \left (\frac{p(\mathbf{x}_{i})}{1-p(\mathbf{x}_{i})} \right) = \mu + \sum\limits_{j=1}^{m} z_{ij} {SNP}_{j}  $$

where *p*(**x**_*i*_) is the *P*(*Y*=1|*X*) for individual *i* with vector of predictors **x**_*i*_; *SNP*_*j*_ is the effect of the *j*_*th*_ marker; *z*_*ij*_ is the genotype of individual *i* at locus *j* (0, 1 or 2 for AA, AB and BB genotypes). Equation 3 returns the odds of *p*(*x*) which are backtransformed to *P*(*Y*=1|*X*) through the cumulative distribution function of the logistic distribution (i.e. the logistic function). Individuals with *p*(*x*)>0.5 were classified as carriers of the haplotype, and vice versa.

Equation 3 substituted Equation 1 in the algorithm 2 and was run in the same cross-validation scheme yielding (100 folds, 100 repetitions, 6 proportions of SNPs) 6000 replicates per SNP-chip.

### Software and computation architecture

All statistical analyses were performed using the *R* programming environment. The functions *regsubsets* and *lda* from the *R* packages *leap*s and *MASS* were used for backward stepwise variable selection and linear discriminant analysis, respectively. The function *glm* of base *R* was used to fit logistic regression. The *R* packages *foreach* and *doParallel* were used for coarse-grained parallelization of the code to speed up computation.

The analyses were run on the bioinformatics platform at PTP, which includes a high performance computing cluster with 600 CPUs, 2.5 TB of RAM and 100 TB of data storage for archiving and backup.

## Results

The size of predictive SNP-sets ranged from 207 to 5 and from 699 to 17 with the 7K and 54K SNP-chip, respectively. The number of SNPs that best predicted haplotype carriers for each proportion of SNPs in the procedure (2.5, 10, 15, 30, 50 and 100*%*) are reported in Tables 3 and 4 for the 7K and 54K SNP-chip.

**Table 3:** **Total error rate, false positive and false negative rate for the prediction of haplotype carriers in the training and testing sets using the bovine LD (7k) SNP-chip**

***LD-chip***	**Training set**			**Testing set**		
**# SNPs**	**TER**	**FPR**	**FNR**	**TER**	**FPR**	**FNR**
5	1.55	1.33	2.91	1.59	1.36	3.03
21	0.84	0.69	1.73	0.94	0.79	1.87
31	0.71	0.57	1.57	0.83	0.68	1.77
62	0.59	0.46	1.38	0.81	0.66	1.72
104	0.52	0.40	1.29	0.78	0.61	1.81
207	0.50	0.37	1.27	0.75	0.57	1.82

Results are averages over 6000 cross-validation replicates (10 folds, 6 proportions, 100 repetitions). TER: total error rate; FPR: false positive rate; FNR: false negative rate.

**Table 4:** **Total error rate, false positive and false negative rate for the prediction of haplotype carriers in the training and testing sets using the bovine 54k SNP-chip**

***54K-chip***	**Training set**			**Testing set**		
**# SNPs**	**TER**	**FPR**	**FNR**	**TER**	**FPR**	**FNR**
17	0.38	0.41	0.20	0.46	0.48	0.28
70	0.28	0.32	0.00	0.39	0.43	0.13
105	0.27	0.32	0.00	0.38	0.42	0.15
210	0.24	0.28	0.00	0.40	0.44	0.17
350	0.21	0.25	0.00	0.41	0.45	0.19
699	0.20	0.24	0.00	0.44	0.47	0.28

Results are averages over 6000 cross-validation replicates (10 folds, 6 proportions, 100 repetitions). TER: total error rate; FPR: false positive rate; FNR: false negative rate.

**Figure 1:**
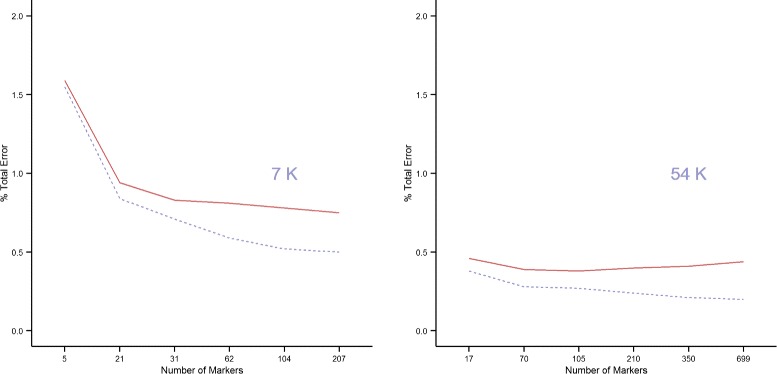
**Total error rate.** Average total error rate (TER) for haplotype carrier identification in the training (grey dotted line) and testing (red solid line) sets; 7K (left) and 54K (right) SNP chips.

Figure 1 shows the total error rate (TER) as a function of the number of predictors (SNPs) in the linear discrimant analysis classification model. TER in the training (dotted line) and testing (solid line) subsets is reported for both the 7K (left) and 54K (right) SNP chips. TER decreases continuously in the training set (from 1.55*%* to 0.50*%* and from 0.38*%* to 0.20*%* with the 7K and 54K SNP-chip, respectively). However, the test error rate is on average higher than the training error rate (0.15 percentage points with both the 7K and 54K SNP chips), and appears to reach a minimum thereafter either stabilising (7K SNP-chip) or beginning to increase again (54K SNP-chip).

It is often interesting to look not only at the total error rate, but also at the error rates within the two groups: haplotype carriers and non-carriers (see Tables 3 and 4). These are usually referred to as the false positive (true non-carriers predicted as carriers) and false negative (true carriers predicted as non-carriers) error rates (FPR, FNR). False negative carriers tended to be more frequent than false positive carriers when using the 7K SNP-chip (test FNR on average 1.23 percentage points higher than the test FPR). Interestingly, with the 54K chip it was the other way around: the test FNR was on average 0.25 percentage points lower than the test FPR).

## Discussion

In this paper, a general procedure for the identification of carriers of a given haplotype, mutation or gene allele from SNP genotypes in farm animals is described. The interest lies in the combination of a variable selection algorithm and a classification method to accurately predict carrier status, rather than in the specific haplotype that was chosen for illustration. The procedure is in principle applicable to any diploid organism (animals, plants, humans).

On average, a very low error rate for the prediction of haplotype carriers was estimated in this study under all models (average total test error rate ∼≤1*%*). Low error rates for allele prediction at the HLA -humans (∼0−5*%*)- and casein -cattle (∼6*%*)- loci were reported in previous studies [11,19]. SNP genotypes are expected to be good predictors for genomic sequences (haplotypes, gene alleles) and a high prediction accuracy can therefore be reasonably achieved. The results presented here are in line with those in the literature, and confirm that this is a highly effective approach bound to achieve a predictive ability close to 100*%*, thus making it a potentially very effective tool for practical applications.

Some relevant aspects of the methodology and results are discussed below. Our procedure aims at classifying individuals -based on their SNP genotypes- in two classes, which in the chosen example are carriers or not of the BH2 haplotype. This does not preclude that the underlying causative mutation may be in high linkage disequilibrium (as is the case for the JH1 haplotype and the CWC15 gene in Jersey cattle) or not (as the mutation responsible for CVM in Holstein cattle) with the BH2 haplotype. Indeed, this has an impact on the identification of carriers of the mutation through the haplotype. This is discussed in the paragraph “Extension to another mutation”.

### Cross-validation: the right way

The key objective of this paper was to estimate the error that can be incurred when classifying individuals into two mutually exclusive groups: carriers and non-carriers of a given haplotype, in the chosen example. A valid estimate of the error rate is obtained when the model trained on some experimental data is tested on an independent set of data (not used in the training process). The training and test error rates can be quite different indeed. As the number of predictors increases, the risk of overfitting the data becomes higher, thereby increasing the chance of finding models that look good on the training data but which will have a low predictive ability on independent data. K-fold cross-validation is a powerful method to ensure the validity of the estimated error rate. However, it is crucial that cross-validation is carried out in the “right way” [27]. If cross-validation of statistical models is carried out in the “wrong way”, it can lead to non-reproducible results and underestimation of the prediction error; this can have serious consequences (see for instance genespurportedly reported to be predictive of survival in patients with follicular lymphoma [28]). Especially when *p*>>*n* (many more parameters *p* than observations *n*), it is tempting to preselect predictors based on their relation with the phenotype, and then to apply cross-validation to the reduced dataset.

In this work, our aim was to obtain a valid estimate of the error in identifying haplotype carriers. A 10-fold cross-validation scheme was adopted: observations were assigned to the training and testing sets before the variable selection and classification steps. Each fold was in turn included in the training set and used for validation.

### Training and testing error

The training error rate -primarily as a consequence of overfitting the data- tends to be lower than the test error rate and underestimates the true classification error. The error is given by the bias and variance of the classifier: $E(y-\hat {f}(x))^{2}=Var(\hat {f}(x))\, +\,[Bias(\hat {f}(x))]^{2}$. Therefore, besides a more or less accurate fit to the data (“bias”), there will be variability between successive estimates of the error.

**Figure 2:**
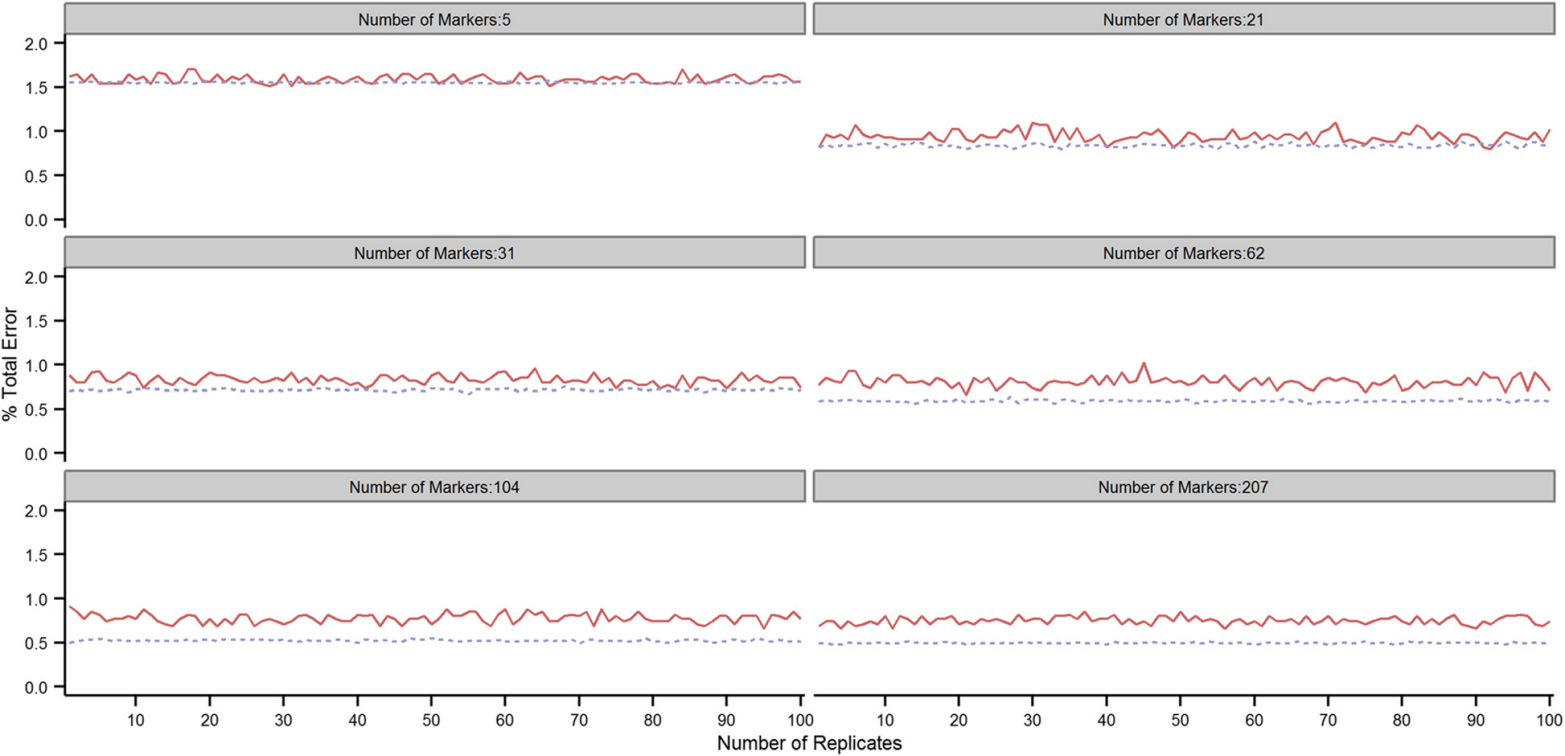
**Error rate across replicates with the 7K SNP-chip.** Total error rate (TER) in the training (grey dotted line) and testing (red solid line) sets for each of the 100 repetitions of the 10-fold cross-validation with increasing proportions of SNPs (2.5, 10, 15, 30, 50 and 100*%*) included in the model when using the 7K SNP-chip.

**Figure 3:**
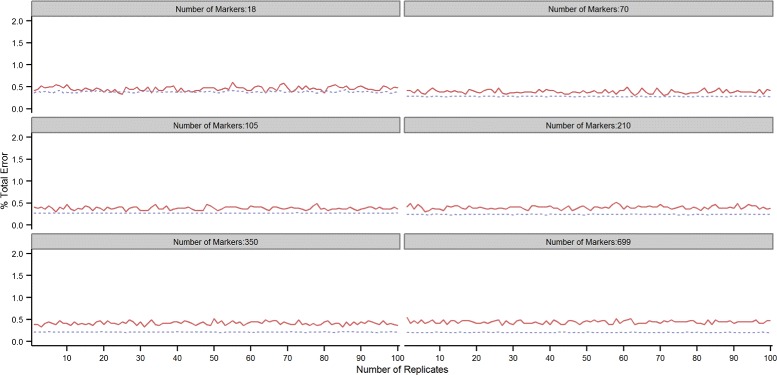
**Error rate across replicates with the 54K SNP-chip.** Total error rate (TER) in the training (grey dotted line) and testing (red solid line) sets for each of the 100 repetitions of the 10-fold cross-validation with increasing proportions of SNPs (2.5, 10, 15, 30, 50 and 100*%*) included in the model when using the 54K SNP-chip.

This can be seen in Figures 2 and 3 which show the test (red solid line) and training (grey dotted line) error rates over the 100 repetitions of the 10-fold cross-validation scheme for the 7K and 54K SNP chips. At each repetition, the average training and test error rates over the 10-fold CV replicates were computed. Although the training error is generally lower than the test error, the opposite can sometimes occur, unexpectedly leading to a lower error in the test than in the training set. The two lines -training and test error- intersect relatively often when small proportions of SNPs are used in the predictive model. This is especially true with the 7K SNP-chip: 27 and 5 times out of 100, the cross-validated test error was lower than the training error when using respectively 2.5 and 10*%* of the SNPs. With the 54K SNP-chip, the test error was lower than the training error 7 times out of 100 only when using 2.5*%* of the SNPs. At higher SNP densities, the test error was always higher than the training error with both SNP chips. Training and test error rates tended to progressively drift farther apart with increasing SNP density. This highlights the importance of resampling in order to have multiple validation sets, particularly at low SNP densities.

### SNP density

Two SNP-chips with different marker densities were evaluated in this study for their predictive ability in *Bos taurus*: the 7K and 54K SNP chips. The two chips differ in number (211 vs 1512) and density of SNPs on BTA19.

Haplotype carriers could be best identified with the 54K SNP-chip: the total error rate and the false positive and false negative rates were all lower compared to those obtained with the 7K SNP-chip (averages over the six proportions of SNPs included in the model: 0.41, 0.44 and 0.24 vs 0.95, 0.77 and 2.00). This was not surprising, considering that the 54K is more informative than the 7K chip, since it contains more SNPs that are closer to the haplotype.

The two SNP-chips showed a different behaviour with respect to the number of false positive and false negative carriers. With the 7K SNP-chip, the test FNR was always higher than the test FPR, while the opposite was true with the 54K SNP-chip. In other words, the 54K SNP-chip appears to have higher “statistical power”, and is relatively better able to identify true carriers of the haplotype. This might be linked to the fact that, compared to the 7K chip, in the 54K chip there are more SNPs in the region of the haplotype, which are likely to be in stronger linkage disequilibrium (LD) with it. The degree of LD in a genomic region is in fact directly related to marker density [29]: with lower marker densities, LD patterns in the bovine genome are only poorly estimated, and higher SNP densities are required for LD patterns to emerge and for SNPs to be in moderate to high LD with genes or haplotypes in the region [30].

The haplotype used in this work has been reported to lie between 10.140 and 11.049 Mb on BTA19 [23]. The average estimated LD between adjacent markers in the region of the haplotype -measured as *r*^2^- was 0.107 with the 7K chip and 0.126 with the 54K chip, which suggests that LD between the SNPs and the haplotype to be identified may account for different predicting performances with different SNP densities.

### Dimensionality of the problem

The procedure outlined in this paper can be applied directly to problems where *n*>*p*, i.e. the number of data points exceeds the number of parameters to be estimated. When there are more parameters than observations, especially if *p*>>*n*, different approaches are needed. Backward stepwise selection (BSS) of variables, for instance, requires that the number of observations *n* is larger than the number of predictors *p*, so that the full model can be fit using least squares. BSS, in fact, starts with the full model containing all predictors, and then goes backwards removing one predictor at a time, until the best fitting model is obtained. If *p*>*n*, this approach would clearly not work and a different variable selection procedure would have to be used: for example, forward step-wise selection [25], which begins with a reduced model containing only one predictor and then progressively keeps on adding predictors until the best model is found. Alternatively, a different method to fit the model should be adopted, such as penalized likelihood [31], that can handle high dimensional problems and avoid inconveniences due to unidentifiable parameters.

Also standard classification methods, such as linear discriminant analysis, are bound to result in no solution at all or unstable results [32] when *p*>*n*. When this is the case, redundant variables may be present in the data, that contribute no additional information to the problem at hand, and issues with linear dependencies might emerge. To circumvent such difficulties, either the dimension of the problem is reduced (e.g. by deleting some variables or by computing *M*<*p* linear combinations of the original variables, like in principal components regression), or some regularization techniques are applied, such as L1-norm (e.g. lasso) or L2-norm (e.g. ridge regression) regularization [20].

### Subset of predicting SNPs

**Figure 4:**
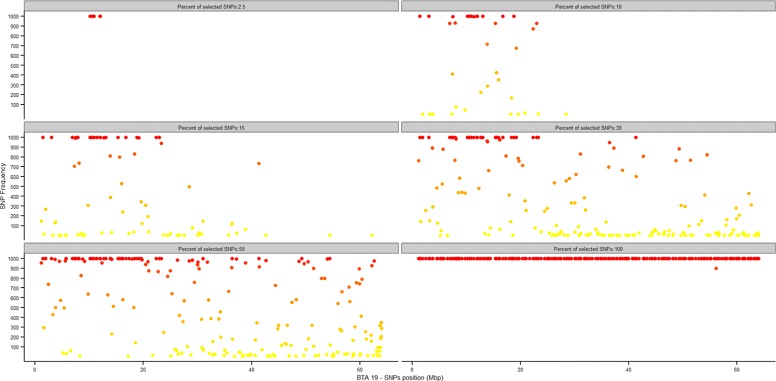
**Predictive SNPs with the 7K SNP-chip.** Frequency of inclusion in the model at each of the 6000 cross-validation replicates for individual SNPs, plotted against their position on BTA19. 7K SNP-chip.

**Figure 5:**
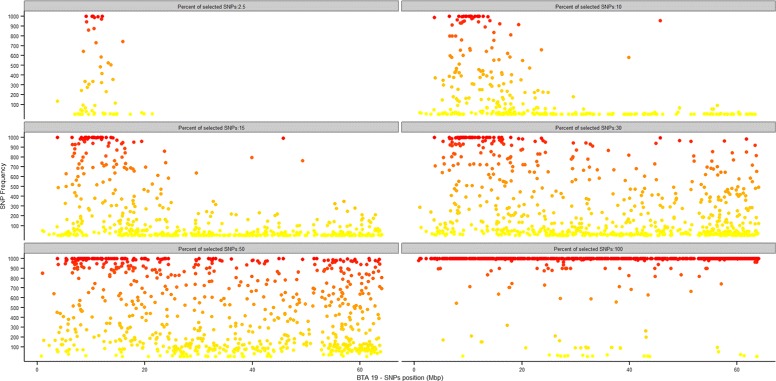
**Predictive SNPs with the 54K SNP-chip.** Frequency of inclusion in the model at each of the 6000 cross-validation replicates for individual SNPs, plotted against their position on BTA19. 54K SNP-chip.

In SNP-based predictive models it can be of interest to identify which SNPs are actually relevant for accurate predictions. When resampling strategies are adopted to obtain a valid estimate of the classification error, the relevance of individual predictors can be indirectly inferred from how often they are present in the different replicates of the model. Figures 4, and 5 report, for the 7K and 54K SNP-chips respectively, the absolute frequency with which each SNP was included in the predictive model, plotted against the position in Mb along BTA19. For each proportion of SNPs to be used for predictions, the number of times each SNP was included in the model in the 1000 test replicates (10-fold CV × 100 repetitions) was counted: dark orange/red and light orange/yellow points in the plot correspond to SNPs most and least frequently used in the predictive models. In this way, genomic regions that harbour SNPs most relevant for prediction can be identified. In Figure 4 and, more clearly, in Figure 5 a region at the beginning of BTA19 emerges as relevant for haplotype prediction. This region is known to contain the BH2 haplotype between 10.140 and 11.049 Mb [23]: the signal was detected by analysing results from the classification procedure. This approach, based on several resampling replicates, is likely to be quite robust and could complement genome-wide association studies (GWAS) for categorical traits (e.g. calving ease in cattle, case/control studies). GWAS is in fact known to potentially suffer from some limitations, like susceptibility to spurious associations and poor reproducibility of results [33,34].

### Comparison with logistic regression

The classification step in the linear discriminant analysis of the procedure *HaplotypeCarrierIdentification* (Table 2) can in principle be replaced by any other linear or non-linear classification method (e.g. quadratic discriminant analysis, support vector machines). In the present work, LDA was compared with a logistic regression classifier. Logistic regression models directly the probability *p*(*x*) of belonging to either class given the predictors, whereas linear discriminant analysis models the distribution of predictors separately in each class and then uses Bayes’ theorem to flip these around and obtain *p*(*x*). Logistic regression was tested with both the 54K and 7K SNP chips, and the results for the identification of haplotype carriers were similar to those obtained with LDA. The average test error rate was equal to 1.25 with the 7K SNP-chip and 0.58 with the 54K SNP-chip, which are slightly higher than with LDA. Also the proportions of false positive and false negative carriers were in line with the results of LDA. With both classifiers, the overall test error rate was very low, close to or lower than 1*%*.

### Extension to another mutation

It is usually important to assess the validity of research results, i.e. how well they can actually translate to the real phenomenon that was modeled, and to what extent they can be generalised. The described statistical methodology for the identification of carriers was therefore applied to another mutation of interest in dairy cattle: spinal dysmyelination on BTA11 (SDM, [35]). Three hundred and five Brown Swiss bulls typed for SDM (8 carriers, 297 non-carriers) were available for validation. All bulls had genotypes from the 7K SNP-chip (337 SNPs on BTA11).

The total test error rate for SDM, with different proportions of SNPs in the model, ranged from 1.64*%* to 38.01*%*. The highest prediction accuracy was achieved when the smallest number of SNPs (i.e. 8) was included in the model.

Overall, the procedure proved to be effective even when tested on a different mutation. However, compared to the results for the BH2 haplotype, a higher test error rate was estimated for SDM. In particular, a larger discrepancy due to overfitting was observed between the training (where the error rate approached 0) and the test error rate. This is likely related to the different frequency of carriers in the population: the ratio of carriers relative to non-carriers was 0.164 for BH2 and as low as 0.027 for SDM. Highly unbalanced data are expected to yield worse predictive performances. This was especially true in the case of SDM for which there were only eight carriers. Also, the smaller sample size available for SDM probably played a role in the higher prediction error rates relative to BH2. Finally, BH2 is a haplotype that spans ∼909 kb, whereas SDM is a point mutation: this may have an impact on linkage disequilibrium, phase concordance and likelihood of a recombination event between the SNPs and the genome segment to be predicted. Sonstegard et al. [36] pointed out that the concordance between a haplotype and the associated mutation varies from case to case: it can be as high as 99.3*%* between the JH1 haplotype and the *CWC15* gene in Jersey cattle; or it may be far less reliable if two versions of the associated haplotype exist, one with and one without the causative mutation, as is the case for complex vertebral malformation (CVM) in Holstein cattle. In this latter case, our procedure would likely be less accurate than for haplotype BH2.

### Application to cattle breeding

In cattle breeding, it is important to identify carriers of undesired mutations in order to select them out of the population [37]. In this perspective, false negative may be more critical than false positive carriers: if the mutation is harmful, it is worse to retain a carrier in the breeding population and have it spread the defect, rather than selecting out a non-carrier.

A method to accurately identify carriers is therefore essential. In this paper, a method that is highly accurate, simple to implement and inexpensive was described. Bulls and cows in breeding programmes are often routinely genotyped for parentage verification, estimation of genomic breeding values and a variety of other purposes. It would therefore be very cost-effective to use already available genotype data to identify mutation or haplotype carriers.

The results of the present study showed that even at low SNP densities the prediction accuracy is about 99*%*, although an excess of false negative carriers was observed with the 7K SNP-chip compared to the 54K chip. However, mixed genotyping strategies can be adopted by which most of the population is genotyped with the 7K SNP-chip and only relatively few animals are genotyped with the 54K chip: imputation techniques [38,39] can then be used to obtain the missing genotypes thereby improving prediction accuracy while minimizing costs. Possible imputation errors are expected to have a negligible impact on prediction accuracy, since imputation accuracy in cattle is close to 99*%*. Mixed genotyping strategies are current standard practice in several national dairy selection schemes (e.g. Canada [40]).

Another possibility is to use the identified subset of predictive SNPs on BTA19 to include them, together with SNPs predictive of other traits of interest, in the design of custom SNP-chips for cattle breeding (an example is the Geneseek Genomic Profiler [41]).

The procedure described in this paper can be applied to the identification of carriers of any mutation or haplotype of interest to breeders. Public repositories do exist that report lists of carriers for specific haplotypes or mutations in cattle, like BH2 [42]. These animals are usually genotyped -being top sires or dams- and could therefore be used as training population for the predictive model in order to identify carriers among genotyped animals with unknown status.

## Conclusions

This paper describes a procedure to select relevant SNPs for the identification of carriers of a given haplotype. A haplotype associated with perinatal mortality in cattle was chosen as illustration. The procedure combined the selection of progressively smaller sets of SNPs and the classification of animals into carriers and non-carriers, thus allowing to assess the predictive ability of the model as a function of the number of SNPs. Very few misclassifications were observed for all subsets of SNPs. The total test error was about 1*%* or lower with both the 7K or the 54K bovine SNP-chips. The test error rate was only slightly higher -especially the false negative rate- when using the lower density SNP-chip. Such high accuracy, together with genotype imputation techniques to optimise genotyping strategies, could potentially make this a very effective and efficient tool for the identification of carriers of any mutation or haplotype of interest in *Bos taurus*. The presented procedure could in principle be extended to any other diploid organism, for agriculture applications in farm animals, crops and trees, and for medical applications in humans.
